# Neuropsychological Outcome following Resuscitation after Out-of-Hospital Cardiac Arrest: A One-Year Follow-Up

**DOI:** 10.1155/2017/7283606

**Published:** 2017-08-06

**Authors:** Jeannette Overbeck, Frank-Michael Schweers

**Affiliations:** ^1^Department of Neurology, Christophorus-Kliniken Dülmen, Vollenstr. 10, 48249 Dülmen, Germany; ^2^Department of Cardiology, Christophorus-Kliniken Dülmen, Vollenstr. 10, 48249 Dülmen, Germany

## Abstract

A 61-year-old woman survived resuscitation after out-of-hospital cardiac arrest. The heterogeneity of the resulting cognitive impairments and the recovery over a one-year period are presented, highlighting the need for standardized neuropsychological testing even after short cardiac arrests and for effective treatment both out of hospital and in hospital.

## 1. Introduction

Whereas about 22% of patients in the USA survive an out-of-hospital resuscitation [1, 2] with a 1.7-fold increase between 2006 and 2010, there are much less survivors in Germany [3]. Success of resuscitation depends on the space of time until onset of revivification and on the efficiency of the techniques applied [4, 5]. Only 15% of German bystanders intervene using cardiopulmonary resuscitation (CPR) [6] compared to 60% in other countries [7].

Short cardiac arrests of at least 10 minutes cause brain damage in regions with high neuronal vulnerability. The reduction of cerebral blood flow in peripheral regions (three-territory border), the need for oxygen of the neocortex, and rapid and delayed neuronal death caused by hyperstimulation of NMDA-receptors are relevant pathophysiological principles for neuropsychological outcomes [8]. Knowledge about cognitive outcomes is still limited nowadays and only about 5% of patients reach their premorbid cognitive base level one month after resuscitation [9]. About 20% to 60% suffer from long-lasting cognitive impairments. Mostly memory, attention, executive functions, and psychomotor functions are affected after cerebral hypoxia [10–12]. Patients present with heterogenous patterns of impairment [13–15].

Recovery of functional impairments after a cardiac arrest continues for months, best progress occurring within the first six months [16]. Improvements of cognitive sequelae are described in the first three months after cardiac arrest with only marginal further improvements until 12 months after the incident [15, 17–19].

## 2. Case Report

A 61-year-old female suffered from cardiac arrest at home. Her husband started initial resuscitation in sense of CPR by bystander until arrival of the emergency physician. After the arrival of the physician ventricular fibrillation was treated with defibrillation. A resulting asystolia was changed into ventricular tachycardia applying norepinephrine. Adding amiodarone, a sinus rhythm was gained and kept stable. On arrival at the clinic mask breathing was changed into endotracheal intubation. Mild therapeutic hypothermia (MTH) was initiated, lasting for 24 hours. The patient was extubated after 48 hours. Electrocardiogram presented a stable sinus rhythm without ST-segment deviation or channelopathies (Brugada syndrome, early repolarization syndrome, and long QT syndrome). The echocardiography showed a nearly unimpaired left ventricular contractile function. Neither a pericardial effusion nor a valvular heart defect could be found.

The patient was investigated with head computed tomography (CT) scan because of anisocoria. Cerebral hemorrhage could be excluded. A thoracic CT scan showed neither pulmonary embolism nor aortic dissection. An obstructive coronary artery disease has been excluded by a coronary angiography. A neurological examination revealed problems of temporal orientation.

Since the anamnesis did not reveal any cardiac illness and the actual physical examinations did not show a cardiomyopathy, an idiopathic ventricular fibrillation (IVF) was diagnosed. Since IVF has a high recurrence rate and drug treatment is ineffective [20], an implantable cardioverter-defibrillator (ICD) was placed. This medical approach is based on the guidelines of secondary prevention of sudden cardiac death.

## 3. Neuropsychological Assessment and Statistics

The patient underwent a neuropsychological assessment which is usually applied in our clinical setting. Considering the guidelines of the German Neurological Society (DGN) [21] examinations of memory should record immediate span, working memory, and delayed recall of both verbal and visual information, as well as a learning paradigm. Attention should be recorded in different terms of selectivity and intensity [22]. Executive functioning is recommended to be checked in at least four ways: working memory, monitoring, cognitive fluency, and flexibility as well as planning and problem solving [23]. The tests used are commonly applied in clinical practice. They are adapted and standardized for the German language area, fulfilling required psychometric standards. The assessment has been conducted shortly before ICD was implanted as well as six and twelve months after the implantation. To examine intrapersonal effects the critical difference [24–26] was computed. The level of significance was set at *p* = .05, one-sided (raw data, Table 1).

### 3.1. Memory

#### 3.1.1. Verbal Memory

Verbal memory examination (VLMT, versions A, C, and D) covered immediate memory span, short-term and long-term retention (free recall and recognition), and learning capacity [27]. The VLMT is a German version of the Rey Auditory Verbal Learning Test.

Immediate span was not affected at all. The patient underachieved in terms of short-term and long-term retention (recall and recognition) at the first trial. At the half-year follow-up all scores revealed a significant improvement, which stayed stable until the one-year follow-up.

#### 3.1.2. Visual Memory

Short-term memory was checked by a block-tapping test (WMS-R) [28] comparable to the Corsi block-tapping task, forward and backward, long-term retention by the delayed reproduction of the Rey complex figure [29], and parallel versions (modified Taylor figure [30], MCG Complex figure 2 [31]). A significant improvement of performance from the baseline to the half-year follow-up in both conditions of the block-tapping test was observed, whereas the forward condition revealed a further significant increase after 12 months. Though long-term retention showed no impairment at the baseline, there was a significant increase of performance after 6 months persisting until the one-year follow-up.

### 3.2. Attention

Attention was recorded by different subtests of a computerized attentive test battery (TAP) [32]. “Alertness” and “Vigilance” were the subtests used for measuring intensity. “Divided attention” and “Go/NoGo” were the subtests used for measuring selectivity. Furthermore, the first two tables of the colour-word-test [33] reflect a dimension of intensity (speed of information processing).

Stimulus selection and reaction inhibition (“Go/NoGo”) were unimpaired at all times of measurement. Divided attention (missing items) showed no improvement after 6 months but after 12 months. Medians of reaction time in phasic and tonic alertness decreased significantly from baseline testing to the half-year follow-up, remaining stable until the one-year follow-up. So did the time for reading and naming colours (colour-word-test, NAI, tables I and II). The patient was not able to run through the vigilance test at all in the beginning. Six months later she had to quit after 15 minutes. After further six months, she reached an average level of performance.

### 3.3. Executive Functions

Working memory and monitoring were recorded by using memory tests (see above) and the colour-word-test, NAI [33], which is comparable to the Stroop-Test. Planning and problem solving were tested by the German version of the Tower of London (TL-D) [34] and verbal and figural fluency by the Regensburg word fluency test (RWT) [35] and, respectively, by a revised version of the Hamasch 5-point test (H5PT-R) [36]. Action flexibility was measured by a programmed motor task (three-step Luria test) [37]. Additionally verbal concept formation was examined by explaining proverbs and differences in word pairs (subtest of SIDAM, structured interview for diagnosing dementia) [38].

Verbal concept formation, planning and problem solving, semantic and phonemic verbal fluency and shifting, and action flexibility were unimpaired throughout the time of record. Figural fluency improved significantly within the first 6 months, persisting on this level until the one-year follow-up. The same applied to the interference effect (reading time for incongruent stimuli, colour-word-test NAI, table III, table III-II).

### 3.4. Constructional Performance

Constructional performance was measured by the Rey complex figure [29] and parallel versions (modified Taylor Complex Figure, MCG Complex Figure 2) [30, 31]. The baseline showed no impairment, neither did the follow-ups.

### 3.5. Language

Confrontative Naming (Boston Naming Test) [39] was not affected at all. This outcome is in line with current literature as speech has been found to be one of the most resistant functions when it comes to cognitive impairments after out-of-hospital cardiac arrest [40].

### 3.6. Orientation

Temporal orientation was completely regained at the first testing time.

### 3.7. Quality of Life

Quality of life was measured by a questionnaire concerning the state of health (SF-36) [41] and revealed no relevant impairments after six and twelve months.

## 4. Discussion

As mentioned before, patients suffering from neuropsychological sequelae show inhomogeneous patterns of cognitive impairments. Deficits in memory are reported in the majority of studies [9, 10, 12, 19, 42] and could be identified in this case as well. Both verbal and visual memory decreased. In particular verbal short-time and long-time retention as well as visual short-time memory have been effected. Studies revealing no other cognitive disturbances often used small test batteries, overemphasizing memory functions [15]. Hence, we applied a set of tests including different aspects of attention, executive functions, visuospatial performance, and language besides memory functions. Our patient underachieved in tonic and phasic alertness, divided attention, vigilance, naming colours, and figural fluency and under interfering conditions. Analogue to physical recovery and congruent to hitherto research of cognitive impairment a lot of functions showed a significant improvement after six months [19, 43, 44]. In contrast to these studies, significant advancements in the forward condition of the block-tapping test and in visual long-term retention and vigilance were observed after twelve months. Cognitive impairments did not influence quality of life during the whole observation period. Referring to Khot and Tirschwell [45] and Ørbo et al. [11], we suppose that there are some reasons for this favourable outcome. To begin with, the elapsed time until onset of bystander-initiated CPR has been extremely short. CPR prior to arrival of the ambulance is assumed to lead to better cognitive functioning [43, 44]. Second, the emergency response time was short. Third, ventricular fibrillation was treated with MTH, which is hypothesized to preserve global brain function and long-term functioning in cardiac arrest survivors [46–48]. Fourth, the patient had no other preexisting risk factors for cognitive problems [19]. Based on the assumption that brain damage has been restricted only to the selectively vulnerable brain regions, improvements beyond the first three months might have been explained with regard to Lim et al.'s (2004) findings. This study underlines the need for effective treatment in and out of hospital, with special emphasis on the need to increase knowledge of basic life support by public education to diminish the number of bystanders failing to come to action. Based on a neuropsychological perspective, this study emphasizes the necessity of cognitive assessments including different aspects of attention, memory, and executive functioning, as well as orientation and constructional performance of patients surviving even short cardiac arrests as a standard procedure to enable rehabilitation if deficits persist. The application of short screenings or, worse, the lack of neuropsychological investigations does not come up to the complexity of information processing. In our case the neurological examination, for example, only revealed problems of temporal orientation.

Further investigations might reveal more details about the relationship between premorbid risk factors, extent of brain damage, medical treatment, and neuropsychological outcome.

## Figures and Tables

**Table 1:** Results at the three measuring points (raw scores).

Skill	Aspect	Measure	Baseline	6 months	12 months
Memory	Verbal immediate span	Correct answers	4	6	6
	Verbal working memory	Correct answers	1	7	7
	Verbal delayed free recall	Correct answers	1	7	7
	Verbal delayed recognition	Correct answers	3	13	11
	Visual memory/immediate span	Correct answers	7	9	12
	Visual memory/working memory	Correct answers	5	10	8
	Visual memory/long-term retention	Points for correctly reproduced items	10,5	17,5	18

Attention	Tonic alertness	Median reaction time in ms	537	316	296
	Phasic alertness	Median reaction time in ms	663	432	322
	Information processing speed/Reading colour names	Seconds	245	18	18
	Information processing speed/naming colours	Seconds	325	23	22
	Divided attention	Missing items	15	6	4
	Vigilance	Missing items/mistakes	Withdrawal	Withdrawal	3/1
	Go/NoGo	Mistakes	1	1	2

Executive functions	Planning and problem solving	Number of correctly solved trials	17	18	17
	Semantic fluency	Number of correct words	13	13	14
	Semantic shifting	Number of correct words	8	13	13
	Phonemic fluency	Number of correct words	8	7	8
	Phonemic shifting	Number of correct words	8	11	10
	Figural fluency	Number of correct figures created	11	24	20
	Interference, table 3	Reading time in seconds	84	43	37
	Interference, table 3-table 2	Difference in seconds	52	20	15
	Verbal concept formation	Correct answers (max. 3)	3	3	3
	Action flexibility	Correct sequence	Yes	Yes	Yes

Visuoconstruction	Copy of complex figure	Correct details (max. 36)	36	36	35

## References

[1] Moulaert V. R. M. P., Verbunt J. A., van Heugten C. M. (2007). Activity and life after survival of a cardiac arrest (ALASCA) and the effectiveness of an early intervention service: Design of a randomised controlled trial. *BMC Cardiovascular Disorders*.

[2] Daya M. R., Schmicker R. H., Zive D. M. (2015). Out-of-hospital cardiac arrest survival improving over time: Results from the Resuscitation Outcomes Consortium (ROC). *Resuscitation*.

[3] Gräsner J.-T., Geldner G., Werner C. (2014). Optimierung der Reanimationsversorgung in Deutschland. *Notfall + Rettungsmedizin*.

[4] Grubb N. R., O'Carroll R., Cobbe S. M., Sirel J., Fox K. A. A. (1996). Chronic memory impairment after cardiac arrest outside hospital. *British Medical Journal*.

[5] Fischer M., Fischer N. J., Schüttler J. (1997). One-year survival after out-of-hospital cardiac arrest in Bonn city: Outcome report according to the ‘Utstein style’. *Resuscitation*.

[6] Gräsner J.-T., Wnent J., Gräsner I., Seewald S., Fischer M., Jantzen T. (2012). Influence of basic bystander resuscitation measures on survival after sudden cardiac arrest. *Notfall + Rettungsmedizin*.

[7] Schneider A., Böttiger B. W., Popp E. (2009). Cerebral resuscitation after cardiocirculatory arrest. *Anesthesia and Analgesia*.

[8] Prohl J., Hundt B., Bodenburg S. (2010). Hypoxisch-ischämische Enzephalopathie (HIE) nach Herz-Kreislaufstillstand (HKS) – Pathophysiologie, Prognose und Outcome eines, vernachlässigten ‘Krankheitsbildes’. *Zeitschrift für Neuropsychologie*.

[9] Moulaert V. R. M. P., Verbunt J. A., van Heugten C. M., Wade D. T. (2009). Cognitive impairments in survivors of out-of-hospital cardiac arrest: a systematic review. *Resuscitation*.

[10] Caine D., Watson J. D. G. (2000). Neuropsychological and neuropathological sequelae of cerebral anoxia: A critical review. *Journal of the International Neuropsychological Society*.

[11] Ørbo M., Aslaksen P. M., Larsby K. (2014). Determinants of cognitive outcome in survivors of out-of-hospital cardiac arrest. *Resuscitation*.

[12] Alexander M. P., Lafleche G., Schnyer D., Lim C., Verfaellie M. (2011). Cognitive and functional outcome after out of hospital cardiac arrest. *Journal of the International Neuropsychological Society*.

[13] Wilson B. A. (1996). Cognitive functioning of adult survivors of cerebral hypoxia. *Brain Injury*.

[14] Armengol C. G. (2000). Acute oxygen deprivation: Neuropsychological profiles and implications for rehabilitation. *Brain Injury*.

[15] Lim C., Alexander M. P., LaFleche G., Schnyer D. M., Verfaellie M. (2004). The neurological and cognitive sequelae of cardiac arrest. *Neurology*.

[16] Raina K. D., Rittenberger J. C., Holm M. B., Callaway C. W. (2015). Functional outcomes: one year after a cardiac arrest. *BioMed Research International*.

[17] Sauve M. J., Doolittle N., Walker J. A., Paul S. M., Scheinman M. M. (1996). Factors associated with cognitive recovery after cardiopulmonary resuscitation. *American Journal of Critical Care*.

[18] Tiainen M., Poutiainen E., Kovala T., Takkunen O., Häppölä O., Roine R. O. (2007). Cognitive and neurophysiological outcome of cardiac arrest survivors treated with therapeutic hypothermia. *Stroke*.

[19] Ørbo M., Aslaksen P. M., Larsby K., Schäfer C., Tande P. M., Anke A. (2016). Alterations in cognitive outcome between 3 and 12 months in survivors of out-of-hospital cardiac arrest. *Resuscitation*.

[20] Tilz R. R., Fedele L., Satomi K., Kuck K. H., Antz M. (2007). Idiopathic ventricular fibrillation. *Herz*.

[21] LL 94 2012 Diagnostik und Therapie von Gedächtnisstörungen. https://www.dgn.org/leitlinien/aktualisierungen.

[22] LL 93 2012 Diagnostik und Therapie von Aufmerksamkeitsstörungen bei neurologischen Erkrankungen. https://www.dgn.org/leitlinien/aktualisierungen.

[23] LL 95 2012 Diagnostik und Therapie von exekutiven Dysfunktionen bei neurologischen Erkrankungen. https://www.dgn.org/leitlinien/aktualisierungen.

[24] Lienert G. (1969). *Testaufbau und Testanalyse*.

[25] Chelune G. J., Naugle R. I., Lüders H., Sedlak J., Awad I. A. (1993). Individual change after epilepsy surgery: practice effects and base-rate information. *Neuropsychology*.

[26] Ringendahl H. (2013). Neuropsychologische Verlaufsdiagnostik bei Parkinson-Patienten – Darstellung kritischer Test-Retestdifferenzen für die Einzelfalldiagnostik. *Zeitschrift für Neuropsychologie*.

[27] Lux S., Helmstaedter C., Elger C. E. (1999). Normative study on the ‘Verbaler Lern- Und Merkfähigkeitstest’ (VLMT). *Diagnostica*.

[28] Härting C., Markowitsch H. J., Neufeld H. (2000). *Wechsler Gedächtnistest – Revidierte Fassung*.

[29] Spreen O., Strauss E. (1998). *A Compendium of Neuropsychological Tests*.

[30] Casarotti A., Papagno C., Zarino B. (2014). Modified Taylor Complex Figure: Normative data from 290 adults. *Journal of Neuropsychology*.

[31] Ingram F., Soukup V. M., Fishel Ingram P. T. (1997). The Medical College of Georgia complex figures: Reliability and preliminary normative data using an intentional learning paradigm in older adults. *Neuropsychiatry, Neuropsychology and Behavioral Neurology*.

[32] Zimmermann P., Fimm B. (2007). *TAP Testbatterie zur Aufmerksamkeitsprüfung, Version 2.1*.

[33] Oswald W. D., Fleischmann U. M. (1997). *Nürnberger Alters-Inventar (NAI)*.

[34] Tucha O., Lange K. W. (2004). *Turm von London – Deutsche Version*.

[35] Aschenbrenner S., Tucha O., Lange K. W. (2000). *RWT Regensburger Wortflüssigkeits-Test*.

[36] Haid T., Martl C., Schubert F., Wenzl M., Kofler M., Saltuari L. (2002). HAMASCH 5 Punkt Test. *erste Normierungsergebnisse. Zeitschrift für Neuropsychologie*.

[37] Weiner M. F., Hynan L. S., Rossetti H., Falkowski J. (2011). Luria's three-step test: What is it and what does it tell us?. *International Psychogeriatrics*.

[38] Zaudig M., Hiller W. (1996). *SIDAM – Handbuch*.

[39] Luck T., Riedel-Heller S. G., Wiese B. (2009). CERAD-NP battery: Age-, gender- and education-specific reference values for selected subtests. Results of the German study on ageing, cognition and dementia in primary care patients (AgeCoDe). *Zeitschrift fur Gerontologie und Geriatrie*.

[40] Roine R. O., Kajaste S., Kaste M. (1993). Neuropsychological sequelae of cardiac arrest. *JAMA*.

[41] Bullinger M., Kirchberger I., Ware J. (1995). Der deutsche SF-36 Health Survey Übersetzung und psychometrische Testung eines krankheitsübergreifenden Instruments zur Erfassung der gesundheitsbezogenen Lebensqualität. *Journal of Public Health*.

[42] Bigler E. D., Alfano M. (1988). Anoxic encephalopathy: Neuroradiological and neuropsychological findings. *Archives of Clinical Neuropsychology*.

[43] van Alem A. P., de Vos R., Schmand B., Koster R. W. (2004). Cognitive impairment in survivors of out-of-hospital cardiac arrest. *American Heart Journal*.

[44] Tiainen M., Poutiainen E., Oksanen T. (2015). Functional outcome, cognition and quality of life after out-of-hospital cardiac arrest and therapeutic hypothermia: Data from a randomized controlled trial. *Scandinavian Journal of Trauma, Resuscitation and Emergency Medicine*.

[45] Khot S., Tirschwell D. L. (2006). Long-term neurological complications after hypoxic-ischemic encephalopathy. *Seminars in Neurology*.

[46] Lim G. H., Seow E. (2002). Resuscitation for patients with out-of-hospital cardiac arrest: Singapore. *Prehospital and Disaster Medicine*.

[47] Kowalik R., Szczerba E., Kołtowski Ł. (2014). Cardiac arrest survivors treated with or without mild therapeutic hypothermia: Performance status and quality of life assessment. *Scandinavian Journal of Trauma, Resuscitation and Emergency Medicine*.

[48] Wachelder E. M., Moulaert V. R. M. P., van Heugten C., Verbunt J. A., Bekkers S. C. A. M., Wade D. T. (2009). Life after survival: Long-term daily functioning and quality of life after an out-of-hospital cardiac arrest. *Resuscitation*.

